# 

**Published:** 1850-06

**Authors:** 


### CONTENTS

OF THE

### MEDICAL EXAMINER.

NEW SERIES—NO. LXVI.—JUNE, 1850.

### ORIGINAL COMMUNICATIONS.

Philadelphia County Medical Society.—Some Hints on the Comparative Influence of dry and moist hot air in the causation of Disease. By Samuel Jackson M. D., (late of Northumberland,) President of the Society, - - - - - - - - - 319

Pulmonary Gangrene. By Moreton Stillé, M. D., of Philadelphia, - 333

A Case of Cyanosis. By William Waters, M. D., Fredericktown, Maryland, - - - - - - - - - - - - - 340

### BIBLIOGRAPHICAL NOTICES.

A Practical Treatise on Inflammation of the Uterus and its appendages, and on Ulceration and Induration of the Neck of the Uterus. By James Henry Bennett, M. D., Member of the Royal College of Physicians; Physician Accoucheur to the Western General Dispensary, &c. &c., - - - - - - - - - - 343

An Essay on the Opium Trade, including a Sketch of its History, Extent, Effects, etc., as carried on in India and China. By Nathan Allen, M. D., - - - - - - - - - - 346

A Systematic Treatise, Historical, Etiological and Practical, on the Principal Diseases of the Interior Valley of North America, as they appear in the Caucasian, African, Indian, and Esquimaux Varieties of its Population. By Daniel Drake, M. D., - - 348

### EDITORIAL.

American Medical Association, - - - - - - - - 357

Assimilated Rank in the Navy, - - - - - - - - 358

Proceedings of the American Medical Association, at the Third Annual Meeting, held at Cincinnati, May, 1850, - - - 359

National Medical Convention for revising the Pharmacopœia of the United States, - - - - - - - - - - 376

University of Pennsylvania, - - - - - - - - 378

